# MicroRNA Roles in the Nuclear Factor Kappa B Signaling Pathway in Cancer

**DOI:** 10.3389/fimmu.2018.00546

**Published:** 2018-03-19

**Authors:** Jin’en Wu, Juntao Ding, Jing Yang, Xiaola Guo, Yadong Zheng

**Affiliations:** ^1^State Key Laboratory of Veterinary Etiological Biology, Key Laboratory of Veterinary Parasitology of Gansu Province, Lanzhou Veterinary Research Institute (CAAS), Lanzhou, China; ^2^College of Life Science and Technology, Xinjiang University, Urumqi, China; ^3^Jiangsu Co-innovation Center for Prevention and Control of Important Animal Infectious Diseases and Zoonoses, College of Veterinary Medicine, Yangzhou University, Yangzhou, China

**Keywords:** microRNAs, nuclear factor kappa B, cancer, molecular therapy, nuclear factor kappa B-associated resistance

## Abstract

Nuclear factor kappa B (NF-κB) is a pluripotent and crucial dimer transcription factor that orchestrates various physiological and pathological processes, especially cell proliferation, inflammation, and cancer development and progression. NF-κB expression is transient and tightly regulated in normal cells, but it is activated in cancer cells. Recently, numerous studies have demonstrated microRNAs (miRNAs) play a vital role in the NF-κB signaling pathway and NF-κB-associated immune responses, radioresistance and drug resistance of cancer, some acting as inhibitors and the others as activators. Although it is still in infancy, targeting NF-κB or the NF-κB signaling pathway by miRNAs is becoming a promising strategy of cancer treatment.

## Introduction

Tumorigenesis is the result of multistep interactions of multi-factors and -genes, and the mechanisms whereby the tumors occur need to be further explored. In recent years, cancer research has undergone a rapid development and achieved a major breakthrough. The 10 hallmarks that are involved in tumor growth and metastasis have been reported (1), and their complex progression ultimately induces incipient tumor cells to develop into tumorigenic and malignant cancer.

MicroRNAs (miRNAs), a class of endogenous and single-stranded RNA, are a subfamily of small non-coding regulatory RNA with a size of 18–22 nt, being involved in various physiological and pathological processes. Increasing studies have demonstrated that miRNAs play a significant role in tumorigenesis and progression, *via* directly or indirectly regulating the expression of various oncogenes or tumor suppressors (2–4). As a transcription factor, nuclear factor kappa B (NF-κB) expression is transient and tightly regulated in normal cells, but it is highly activated in cancer cells (5). NF-κB is not only involved in immune responses (6, 7), but it also plays an important role in the development and progression of tumor (8, 9), metastasis (10), and drug resistance (11). Recently, it has been demonstrated that miRNAs are involved in the regulation of NF-κB signaling pathway by different mechanisms (12–14). These interactions suggest that miRNAs and NF-κB can be used as potential tumor diagnostic biomarkers and drug therapeutic targets in clinical treatment of cancer.

## miRNA Biogenesis and Its Expression in Cancer

MicroRNAs are mainly transcribed by RNA polymerase II, and in the canonical pathway the resulting transcripts, known as primary miRNAs, are cleaved by Drosha-DGCR8 to produce precursor miRNAs (pre-miRNAs) that are exported to the cytoplasm by exportin-5 and Ran-GTP (15, 16). In the cytoplasm, pre-miRNAs are processed into miRNA duplexes by Dicer, and mature miRNAs are incorporated into the AGO2-containing small RNA-induced silencing complex, while the counterparts, known as miRNAs*, are degraded in most cases (2, 17, 18). In addition to the canonical pathway, a small part of miRNAs are derived from introns and spliced by spliceosome in a Drosha-independent pathway (19). miRNAs can regulate mRNA stability or translation by interacting with binding site(s) in the 3′ untranslated region (UTR) of targets.

Increasing literatures have documented that miRNAs are implicated in the pathogenesis of various malignancies (20, 21), and they represent key players in cancer development and metastasis processes as well (22), such as thymic epithelial tumors (23), renal cancer (24), colorectal cancer (25), and so on. It is undoubted that miRNA expression profiles are different in the distinct tumors, and a specific set of miRNAs are upregulated or downregulated in the specific tumor cells (4). Although the correlation between miRNA dysregulation and cancer has been demonstrated, it is still not clear whether changed expression of miRNAs promotes carcinogenesis or the development of cancer causes ectopic expression of miRNAs. Therefore, understanding the functions of miRNAs and their targets in the relevant signaling pathways is of great importance and may help us develop potential diagnosis and therapeutic approaches for cancer.

## NF-κB Signaling Pathway

**Figure 1:**
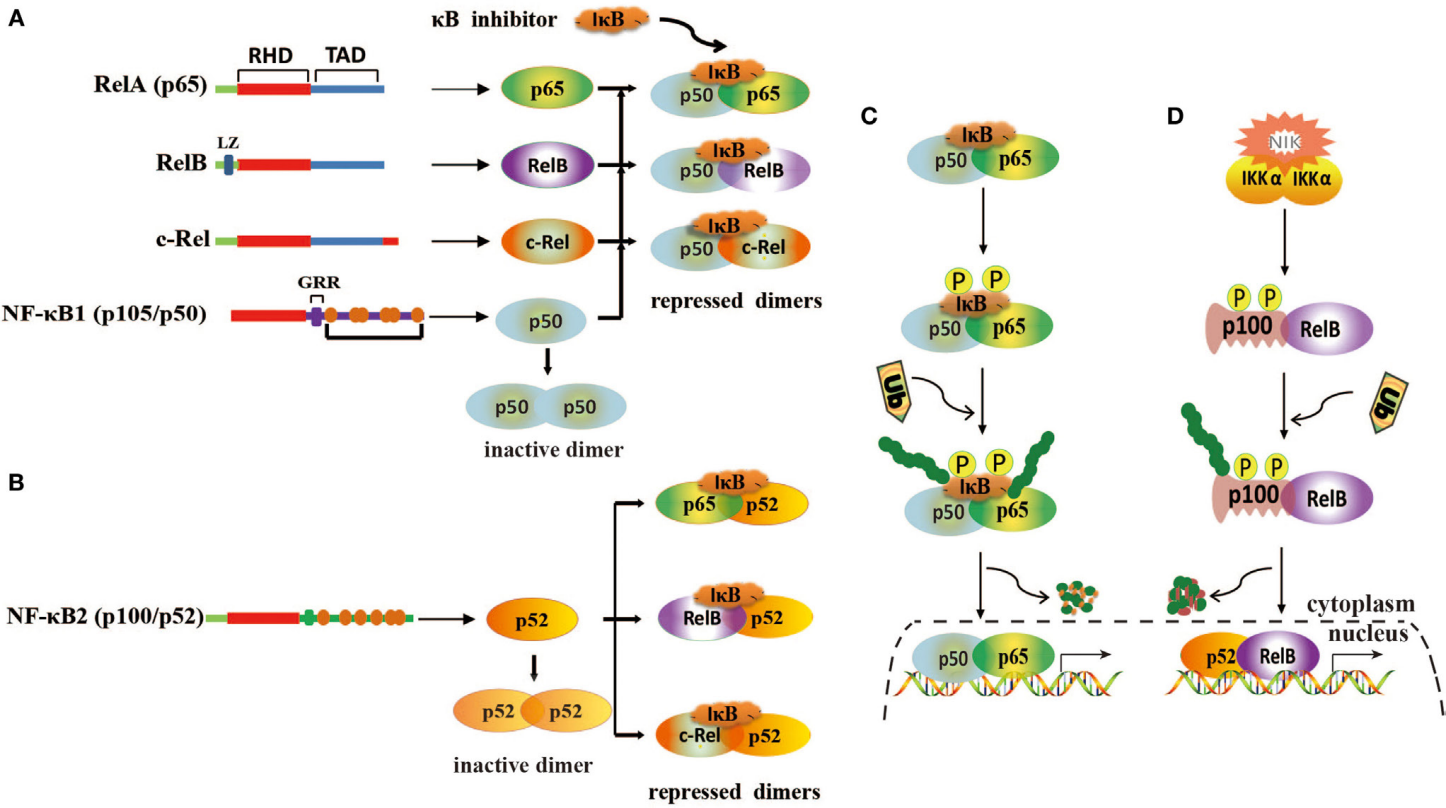
Structure of nuclear factor kappa B (NF-κB) and its activation mechanisms. **(A)** Homodimerization and heterodimerization of NF-κB1. Homodimer p50 lacks transcriptional activation domains and is unable to bind to DNA. Heterodimers are repressed by IκB. **(B)** Homodimerization and heterodimerization of NF-κB2. Homodimer p52 lacks transcriptional activation domains and is unable to bind to DNA. Heterodimers are repressed by IκB. **(C)** Classical pathway of NF-κB activation. This pathway is mediated by IκB kinase complex (IKK), leading to phosphorylation and degradation of IκB. **(D)** Alternative pathway of NF-κB activation. This pathway involves IKKα activation *via* NF-κB-inducing kinase (NIK) and induces the phosphorylation of p100, leading to the generation and translocation of p52.

Nuclear factor kappa B is a transcription factor that presents in the cytoplasm of a cell and regulates the expression of immune and growth genes (26, 27). It is heterodimeric or homodimeric combinations of five different protein subunits, including RelA (p65), RelB, c-Rel, NF-κB1 (p50/p105), and NF-κB2 (p52/p100) (Figures 1A,B), all of which commonly share a N-terminal Rel homology domain responsible for DNA binding and dimerization (28, 29). All 5 family members can potentially form 15 different homodimeric or heterodimeric complexes (30). The transcription activation domain (TAD) is necessary for gene expression, but both NF-κB1 (p50/p105) and NF-κB2 (p52/p100) lack TAD (31). Thus, p50 and p52 cannot activate gene expression unless they associate with a NF-κB family member containing TAD or recruit a specific coactivator (29). In resting cells, NF-κB dimers are sequestered in the cytoplasm through combining inhibitory protein IκB (IκBα/β/γ) (Figures 1A,B). When cells are stimulated by various agents, such as bacteria, virus, cytokines, and tumor promoter, NF-κB is rapidly activated and translocated into the nuclear to promote the expression of genes by binding to κB sites (32). Generally, NF-κB is activated by two signaling pathways, a classical pathway and an alternative pathway (33). In the classical pathway, NF-κB activation is precisely mediated through the phosphorylation and polyubiquitination of IκB members and then degradation by proteasome (Figure 1C) (34). In the alternative pathway, p100 and p105 are processed and degraded to p52 and p50 by an IκB-independent pathway (Figure 1D) (35).

In normal cells, NF-κB is tightly controlled by regulating its inhibitor IκB. However, in cancer cells, NF-κB is highly activated and translocated to the nucleus to induce cell proliferation and immortalization by dysregulation of various signaling pathways (8, 36). These processes are also regulated by miRNAs that modulate the NF-κB signaling pathway.

## Regulation of NF-κB Signaling by miRNAs in Cancer

Nuclear factor kappa B is activated in various cancer types as an important inducible carcinogenesis mediator (37), making malignant tumor cells to evade apoptosis from cell cycle checkpoint (38). In recent years, increasing evidence has demonstrated that miRNAs and NF-κB play an important role in tumor development and progression (6, 39, 40). Particularly, NF-κB can be directly or indirectly activated by miRNAs in cancer cells or oncogenic human virus-infected cells (Table 1).

**Table 1:** MicroRNAs (miRNAs) involved in the regulation of the nuclear factor kappa B (NF-κB) pathway in cancer.

miRNAs	Expression	Cancer type	Target	Function	Reference
miR-30e*	Up	Prostate/gliomas	IκBα	NF-κB activation	(6, 41)
miR-146a/b	Up	Breast/epithelial ovarian/cancer	Tumor necrosis factor receptor-associated factor (TRAF)6/interleukin-1 receptor-associated kinase (IRAK)1/myeloid differentiation factor 88 (MyD88)	NF-κB inhibition	(13, 42, 43)
miR-940	Down	Pancreatic ductal adenocarcinoma	MyD88	NF-κB activation	(44)
miR-514a-3p	Down	Testicular germ cell tumor	Paternally expressed gene 3	NF-κB activation	(40)
miR-133a	Up	Glioblastoma cell	Death receptor 5	NF-κB activation	(45)
miR-223	Up	Lung cancer	IκB kinase complex (IKK)α/β	IκB phosphorylation	(46)
miR-127-5p	Down	Hepatic cellular cancer (HCC)	BLVRB	p65 phosphorylation	(47)
miR-31	Down	Adult T cell leukemia/lymphoma	NF-κB-inducing kinase	NF-κB activation	(48)
miR-26a/b	Down	HCC	TAB 1/transforming growth factor-β-activated kinase 1	IKKβ activation	(49)
miR-15b-5p	Down	Colorectal cancer	IKK-α	IκB activation	(50)
miR-130a	Up	Cervical cancer	Tumor necrosis factor-α	NF-κB inhibition	(51)
miR-429	Down	Cervical cancer/HCC	IKK-β/TRAF6	IκB activation	(52, 53)
miR-k1, -k5, and -k9	Up	Kaposi’s sarcoma-associated herpesvirus-induced lymphoma	IκB-α/IRAK/MyD88	NF-κB activation	(54, 55)
miR-30c-2-3p	Down	Breast cancer	TRADD	NF-κB regulation	(56)
miR-9	Down/up	Ovarian/gastric cancer	NF-κB1	NF-κB activation/suppression	(57, 58)
miR-451	Down	HCC	IKK-β	IκB activation	(59)
miR-218	Up	Glioma cancer	IKK-β	NF-κB inactivation	(60)
miR-210-3p	Up	Prostate cancer	Suppressor of cytokine signaling 1/tumor necrosis factor alpha induced protein 3 interacting protein 1	NF-κB activation	(61)
miR-19b-3p	Up	Nasopharyngeal carcinoma (NPC)	TNFAIP3	NF-κB activation	(62)
miR-125b	Up	NPC	A20	NF-κB activation	(63)
miR-668	Up	Breast cancer	IκBα	NF-κB activation	(64)
miR-20a	Up	Gastric cancer	IκBβ	NF-κB activation	(65)
miR-199a	Down	Ovarian cancer	IKKβ	IκB activation	(66, 67)

### NF-κB Activation by miRNAs in non-Viral Tumors

Numerous pathways can induce the activation of NF-κB in different types of cancer. In addition, miRNAs also target the key components and regulatory proteins of the NF-κB signaling pathway to modulate the activity of NF-κB, such as tumor necrosis factor (TNF), a secreted proinflammatory cytokine. For instance, significantly low expression of miR-9 promoted NF-κB1 overexpression, thus enhancing NF-κB activities and inducing ovarian cancer cell proliferation (57). In cervical cancer cells, upregulated miR-130a directly targeted the 3′ UTR of TNF-α and reduced its expression, and then downregulated TNF-α-activated NF-κB activity and enhanced miR-130a expression by a negative feedback loop of NF-κB/miR-130a/TNF-α/NF-κB (51).

#### miRNAs Involved in the Regulation of Myeloid Differentiation Factor 88 (MyD88)

Myeloid differentiation factor 88 is a critical adaptor protein in the toll-like receptor (TLR)/interleukin (IL)-1R receptor family signaling pathway. It was reported that miR-940 targeted the 3′ UTR of MyD88, being involved in the activation of NF-κB in pancreatic ductal adenocarcinoma (44). A low level of miR-940 led to an elevated expression of MyD88 and promoted pancreatic ductal adenocarcinoma cell growth. Furthermore, miR-146a was also shown to target and reduce MyD88 expression, thus inhibiting the NF-κB activation in epithelial ovarian cancer, and directly regulating the sensitivity of ovarian cancer cells to drug therapy (42). Therefore, miRNAs can regulate NF-κB activities by targeting MyD88, which has a very important significance for tumor development.

#### miRNAs Involved in the Regulation of Tumor Necrosis Factor Receptor-Associated Factor (TRAF)

**Figure 2:**
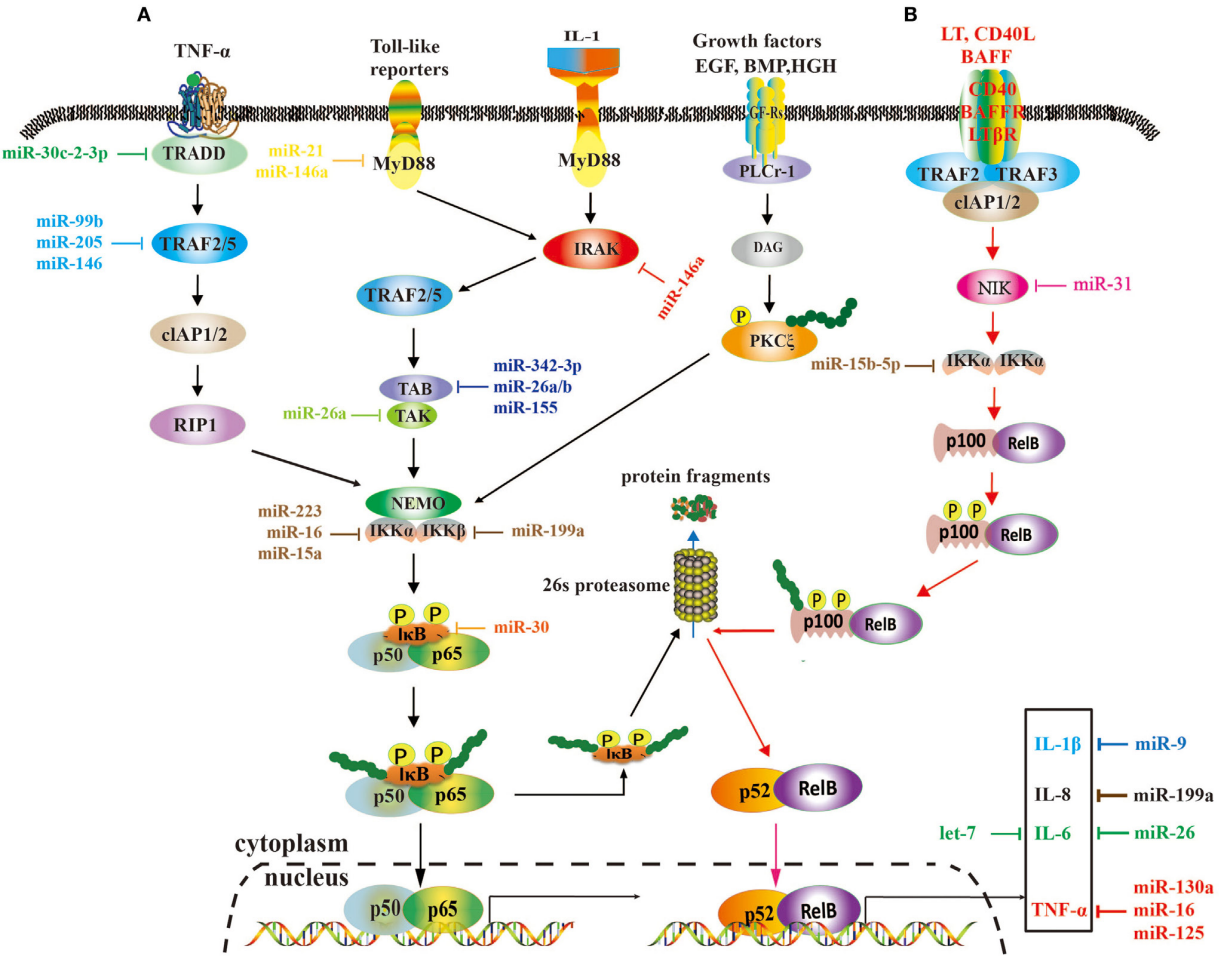
A role of microRNAs (miRNAs) in the nuclear factor kappa B (NF-κB) signaling pathways. These two pathways rely on tumor necrosis factor (TNF), toll-like, interleukin (IL)-1, and EGF receptors **(A)**, and BAFF reporter and CD40 **(B)**, respectively, and they are activated or repressed by miRNAs. Activated NF-κB promotes or restrains the expression of tumor-associated genes.

Tumor necrosis factor receptor-associated factors are a class of multi-functional intracellular signaling adaptor proteins, being involved in signal transduction of multiple receptor families, including apoptosis factor receptor family (TNFR), TLR family (68), interleukin-1 receptor family, NF-κB receptor activating factor family (RANK), and so on (Figure 2). TRAF interacts with downstream proteins in a cell and eventually initiates NF-κB activation through miRNAs. For example, miR-146a/b was identified as an inhibitor for TRAF6 and interleukin-1 receptor-associated kinase (IRAK)1 in breast cancer cells MDA-MB-231 and downregulated TRAF6 eventually gave rise to the suppression of NF-κB activities (43). In human testicular germ cell tumor, loss of miR-514a-3p upregulated paternally expressed gene 3 (PEG3) and consequently overexpressed PEG3 recruited TRAF2 to activate the NF-κB pathway (40). Moreover, it has been recently demonstrated that downregulated miR-429 markedly promoted proliferation and migration of hepatic cellular cancer (HCC) by targeting TRAF6 through the NF-κB pathway, while upregulation of miR-429 significantly suppressed HCC growth (52). Therefore, it is becoming clear that miR-146a/b, miR-514a-3p, and miR-429 may serve as a potential target for the treatment of HCC *via* regulation of TRAF.

#### miRNAs Involved in the Regulation of Transforming Growth Factor-β-Activated Kinase (TAK)1

Transforming growth factor-β-activated kinase 1 is a serine/threonine protein kinase, which is activated by specifically binding to TAB 1–3 protein and ubiquitinated TRAF6, being involving in intracellular signal transduction, Iκβ activation, and NF-κB cascade activation (69). Emerging evidence implies that miRNAs regulate TAK1 expression to promote tumorous invasion, metastasis, and chemoresistance. It was reported that miR-143 targeted TAK1 to attenuate development and progression of pancreatic ductal adenocarcinoma *via* NF-κB pathway (70). Similarly, miR-146a and miR-26b were also demonstrated to target TAK1 to promote gastric cancer cell apoptosis (71) and suppress the NF-κB signaling and enhance the chemosensitivity of HCC (49), respectively. These data suggest that miR-143, miR-146a, and miR-26b act as a potent inhibitor of the NF-κB pathway.

#### miRNAs Involved in the Regulation of IκB Kinase Complex (IKK)

The IKK includes three kinase subunits, one being the regulatory subunit NEMO, also known as IKKγ, and the rest two being serine–threonine kinases (IKKα and IKKβ). They are a signal integration hub for IκB phosphorylation and NF-κB activation (72, 73). Recent studies have showed that abnormal expression of IKK promotes proliferation and invasive ability of cancer cells by miRNAs. miR-218 was able to downregulate IKK-β expression and inactivated the NF-κB signaling, leading to dramatic reduction of the migratory and invasive ability of glioma cells (60). In ovarian cancer, downregulated miR-199a, a regulator of IKK-β expression, promoted a functional TLR–MyD88–NF-κB pathway and induced ovarian cancer cells to secret proinflammatory cytokines and enhance tumor progression (66). As a key tumor suppressor, miR-15b-5p was also markedly downregulated in colorectal cancer. It promoted NF-κB activation through negative regulation of IKK-α that induce IκB phosphorylation (50). In HCC cells, markedly downregulated miR-451 promoted the tumorigenicity by direct targeting of IKK-β. On the contrary, upregulation of miR-451 resulted in downregulation of cyclin D1 and c-Myc through the inhibition of NF-κB pathway, thus decreasing proliferation of HCC cells (59).

#### miRNAs Involved in the Regulation of IκB and Other NF-κB-Associated Components

The activation of NF-κB is universally achieved *via* first IκB phosphorylation by IKKβ and degradation by ubiquitination. In cervical cancer, the downregulated miR-429 elevated IKKβ expression and promoted NF-κB activation by phosphorylating IκBs (53). Nevertheless, miR-30e* was hyperexpressed in prostate cancer and targeted IκBα (61), thus increasing free cytoplasmic NF-κB to translocate into the nucleus to regulate the expression of cyclin D1 and consequently enhancing tumor proliferation and growth (6). Similarly, the expression of miR-210-3p was also elevated in prostate cancer cells, particularly in bone-metastatic prostate cancer cells, and it was directly targeted multiple negative regulators of the NF-κB signaling pathways, including suppressor of cytokine signaling 1 and tumor necrosis factor alpha induced protein 3 interacting protein 1, resulting in constitutive activation of the NF-κB signaling, and promoting EMT of bone-metastatic prostate cancer cells (61). In the lung cancer cells, overexpression of miR-223 was found to significantly promote tumor progression through activating NF-κB, but the mechanisms are still elusive (46). In gastric cancer, miR-20a directly targeted cylindromatosis and IκBβ, promoting activation of the NF-κB pathway and its downstream targets, such as livin and survivin, which potentially induced chemoresistance (65, 74). Moreover, it was reported that overexpression of Polycomb regulated the non-canonical NF-κB pathway by inhibiting miR-31 to upregulate the NF-κB-inducing kinase expression level in adult T cell lymphoma (48). In HCC, downregulated miR-127-5p induced the overexpression of BLVRB to promote NF-κB activity and enhance HCC cell growth (47). Similarly, miR-30c-2-3p also acted as one of the strongest negative regulators and activated NF-κB signaling through downregulation of TRADD in breast cancer (56).

In some cases, the NF-κB signaling pathway can regulate the levels of miRNAs by controlling relative protein expression to participate in cancer occurrence (75). It has been reported that there are four NF-κB-binding sites in the miR-130a gene promoter region and miR-130a is upregulated upon NF-κB binding, which leads to the downregulation of PTEN to promote cervical cancer cell growth (76). The ectopic expression of NF-κB disturbs oncogenic miR-221 and miR-222 expression in prostate carcinoma and glioblastoma cell lines, possibly by NF-κB binding to two sites in the upstream of miR-221/222 promoter (77).

### NF-κB Activation by miRNAs in Viral Tumors

For some oncogenic viruses, their infections can activate the NF-κB signaling pathway by altering cellular endogenous miRNAs. High-risk human papillomavirus (HPV) infection leads to aberrant expression of tumor suppressive miRNAs of infected cells and oncogenicity. HPV E6 and E7 oncoproteins regulate the expression of miR-34a and miR-15a/miR-16-1 *via* degrading tumor suppressor proteins such as p53 and pRB, respectively (78). As a transcription factor, p53 is directly degraded by E6 oncoprotein, which binds to a p53-binding site in the miR-34a promoter region and mediates its expression (79). E7 oncoprotein releases E2F from the pRB–E2F complex and degrades tumor suppressor pRB (80). Free E2F binds to the promoter regions and regulates the expression of miR-15a/miR-16-1 and miR-106b-25 (81). Moreover, several studies have reported that the expression of HPV early protein E1, a viral helicase, activates DNA damage response pathways. Its key regulator, ataxia telangiectasia mutated, has a function in reduction of IκBα by phosphorylation and activation of the NF-κB signaling pathway (82). Afterward, the activated NF-κB binding to the miR-221 promoter region induces its overexpression (83). It is likely that the infection by high-risk types of HPV causes aberrant cellular miRNA expression and then promotes the formation of cervical cancer (78). In addition, upregulation of miR-221 also promotes HCV infection in a NF-κB-dependent manner in HCV-associated HCC (84).

In the other hand, viral miRNAs can also regulate immunoreactions through targeting the NF-κB signaling pathway. Kaposi’s sarcoma-associated herpesvirus miR-k9 and miR-k5 regulated NF-κB activation by targeting IRAK and MyD88 to repress IL-6 and IL-8 expression and enhance viral infection (54). On the contrary, miR-k1 directly repressed the expression of IκBa, enhanced the transcriptional activities of NF-κB, and inhibited viral lytic replication (55). However, these studies on the interactions between viral miRNAs and intracellular NF-κB are still seriously inadequate. Therefore, uncovering NF-κB functions *via* elucidating molecular mechanisms underlying a role of miRNAs in the persistence and pathogenesis of viral infection-induced cancer is extremely important.

## NF-κB-Associated Resistance in Cancer and miRNAs-Targeting Therapy

Chemotherapy, radiotherapy, and targeted therapy are the effective ways for the treatment of tumors, but drug- and radio-resistance inevitably limits therapeutic effects of long term (85, 86). Recently, growing evidence has shown that NF-κB is not only involved in the development and progression of cancer but also exerts the main function in modulating antitumor immunity (87, 88). Meanwhile, it has been reported that the NF-κB signaling pathway can be activated by most chemotherapeutic agents and radiation therapy, and may be a causative factor for drug resistance (89–91). Even for some cancer cells, transient exposure to a low-dose of doxorubicin could enhance drug resistance *via* activation of the NF-κB signaling pathway (92). TRAIL is a promising specifically targeted anticancer agent, but it can stimulate the activation of NF-κB to promote the proliferation of cancer cells (93). Moreover, TRAIL activates a positive feedback loop that sustains the acquired drug resistance by inducing NF-κB-dependent overexpression of miR-21 and miR-100, which both target TRAF7 to further activate the NF-κB signaling (39, 94). Recently, miR-133a was found to be upregulated in the human glioblastoma cell lines M059J and M059K, and it strongly promoted TRAIL resistance by suppressing death receptor 5 expression and activating NF-κB signaling (45). For nasopharyngeal carcinoma (NPC), radiotherapy is the primary treatment strategy, but significantly upregulated miR-19b-3p decreases NPC radiosensitivity by targeting TNFAIP3 to increase NF-κB activity (62). Meanwhile, miR-125b was also found to enhance radioresistance through targeting A20 and then activating the NF-κB in NPC (63). Another example for radioresistance is miR-668 that directly targets the NF-κB inhibitor IκBα to activate NF-κB, and then enhances radioresistance of human breast cancer MCF-7 and T-47D cells (64). Thus, combinatory treatment using anticancer chemotherapy drug and NF-κB inhibitors could revert drug resistance and reduce tumor growth.

Activation of NF-κB affects the transcription of over 400 genes and promotes cells to involve antiapoptotic responses, metastases and drug resistance (28, 95, 96). Targeting NF-κB or the NF-κB signaling pathway by miRNAs will be a promising strategy for the treatment of cancer. For the overexpressed miRNAs regulating the NF-κB signaling, antagonists (anti-miR or antagomiR or antisense oligonucleotides) and modified chemically antisense oligonucleotides against corresponding miRNAs are effective tools to decrease the expression levels of endogenous miRNAs. Antagonists have been demonstrated with a strong potential to effectively downregulate targeted miRNAs in cancer (97). An inhibitor of miR-223, one of the highly expressed miRNAs in lung cancer cells, significantly decreased cell viability and invasion by repressing the expression levels of IKKα/β and NF-κB (46). Overexpression of miR-21, an antionco-miRNA, effectively reduced the proliferation and migration of human colon carcinoma cells. Further studies revealed that elevated miR-21 reduced the phosphorylation of ERK1/2 (98). As phosphorylated ERK1/2 is associated with NF-κB in the nucleus, it is supposed that the NF-κB activity will be decreased in the treated cells. In a diametrically opposite approach, miRNA mimics, a class of artificially synthetic miRNAs, are applied to restore loss of function of miRNAs in cancer cells (99). For example, miR-520b mimics sharply reduced breast cancer cell migration by targeting HBXIP and IL-8 *via* a network, in which HBXIP accelerates cell migration by activating NF-κB-mediated IL-8 expression (100). In ovarian carcinoma cells, the transfection with miR-141 mimics was able to inhibit KEAP1, which can bind to IKKβ to activate the NF-κB signaling pathway, and then enhanced resistance to cisplatin (101). Another therapeutic method is the “miRNA sponge effects.” It refers to robust overexpression of artificial small RNAs using viral vectors such as lentiviruses or adenoviruses encoding miRNA mimics or antagonists, which restore the loss of miRNAs or decrease endogenous miRNAs (102, 103).

It is known that the first miRNA mimics MRX34 has already entered at the clinical treatment phase for the treatment of human pancreatic cancer in 2013 (85). Moreover, both miR-200c EDVs vectors and miR-29b mimics are also at a preclinical stage, and the results have shown that miR-200c enhances radiosensitivity (104) and miR-29b mimics by cationic lipoplexes transfection significantly inhibits tumor growth (105), but their possible long-term side effects should be further analyzed. Therefore, targeting NF-κB might not only directly decrease cancer invasiveness and metastases but also restore tumor cell sensitivity to chemotherapy and radiotherapy. Although miRNAs-targeting therapy attracts more and more attention, dose-dependent toxicity, off-target regulation, biosafety, and so on are still big challenges that need to be solved in the next few years.

## Perspectives

As important transcriptional regulators, miRNAs can upregulate or downregulate many target genes involved in the NF-κB signaling pathway *via* negative or positive feedback loops, which are responsible for cancer initiation, development, progression, metastasis, and drug resistance. Therefore, miRNAs have been applied as molecular therapy targets, diagnosis markers, and prognostic indicators for cancer. Restoration or repression of miRNA expression levels has been showed a high potential for tumor therapy in cells and animal models. However, the clinical applications of miRNAs are greatly limited due to the multi-target, off-target, and instability. Therefore, further studies will be required to confirm the prognostic side effects of miRNA mimics and antagonists.

Furthermore, based on the current knowledge, many studies have just focused narrowly on the specific effects of a given miRNA targeting a specific mRNA by bioinformatic prediction algorithms, rather than exploring the “bigger picture” of gene expression regulation. With the depiction of this “bigger picture,” it is expected that NF-κB-targeting miRNAs are a promising potential target for various cancer treatment.

## Author Contributions

YZ conceived the study; JW, JD, JY, and XG analyzed the data; JW and YZ wrote the paper.

## Conflict of Interest Statement

The authors declare that the research was conducted in the absence of any commercial or financial relationships that could be construed as a potential conflict of interest.

## Abbreviations

NF-κB, nuclear factor kappa B; HCC, hepatic cellular cancer; ATL, adult T cell leukemia/lymphoma; TAD, transcription activation domain; TNF, tumor necrosis factor; RHD, Rel homology domain; TLR, toll-like receptor; IL, interleukin; IRAK, interleukin-1 receptor-associated kinase; PEG3, paternally expressed gene 3; TAK, transforming growth factor-β-activated kinase; SOCS, suppressor of cytokine signaling; TNIP, tumor necrosis factor alpha induced protein 3 interacting protein; CYLD, cylindromatosis.
